# Editorial: Microbial Decontamination by Novel Technologies – Mechanisms and Application Concepts

**DOI:** 10.3389/fmicb.2019.01476

**Published:** 2019-06-28

**Authors:** Alexander Mathys, Kai Reineke, Henry Jäger

**Affiliations:** ^1^Sustainable Food Processing Laboratory, Department of Health Science and Technology, Institute of Food, Nutrition and Health, ETH Zurich, Zurich, Switzerland; ^2^GNT Europa GmbH, Aachen, Germany; ^3^Department of Food Science and Technology, Institute of Food Technology, University of Natural Resources and Life Sciences, Vienna, Austria

**Keywords:** inactivation mechanisms, non-thermal technologies, food safety, minimal processing, process validation

Microbial food safety and effective preservation constitutes the fundamental aspects of global food production systems. Despite of being well-developed in certain domains such as thermal processing; the quality and organoleptic properties of the treated products are limited. Since many years, significant efforts have been made to improve food quality, and in parallel, enabling similar microbial safety levels that of commercial products.

Emerging preservation technology concepts form the core focus of these developments. The main aim of this Research Topic for “Frontiers in Microbiology” is to provide an overview of the recent studies focusing on the inactivation mechanisms during novel non-thermal decontamination treatments and their implication on the development of application and validation concepts.

This Research Topic comprises of 9 original articles (including 2 reviews), contributed by 45 authors.

The fundamental concept and backbone of these research articles is the multi hurdle technology (MHT) concept, suggested by Leistner and Gorris (1995). It includes a broad variety of physical (thermal, electromagnetic, mechanical), physico-chemical, or biological hurdles, where different multi-hurdle combinations lead to targeted additive or even better synergistic effects against microorganisms. The overall goal is the tailored stabilization of individual food products while retaining the beneficial qualities and organoleptic properties.

However, the MHT concept is based on a sensitive balance of specific hurdles for targeted food products or categories. Removing one or more effective hurdles and replacing them by non-thermal technologies is still not well investigated and understood. On this perspective, Schottroff et al. reviewed different non-thermal technologies with a focus on pulsed electric fields, pulsed light, ultraviolet radiation, cold atmospheric pressure plasma, and high isostatic pressure and their cellular as well as molecular mechanisms of action. The authors had focused on sub-lethal injury and the viable but non-culturable (VBNC) states of microorganisms after the novel decontamination treatments. An understanding of these mechanisms based on the advanced analytical measures is essential to ensure effective microbial food safety during storage as well. This becomes more relevant if the inhibition-based hurdles (such as low pH) are replaced with inactivation-focused hurdles, such as high isostatic pressure.

Another review, by Zhang and Mathys, discusses the control of the most resistant form of microorganisms, the bacterial spores. Here, population heterogeneities could occur by different germination and inactivation mechanisms. The so-called super dormant spores could germinate extremely slowly or totally fail to germinate. Although germination is the target of some non-thermal technologies such as high isostatic pressure, understanding the detailed mechanisms of their effects on germination deficiency by proper isolation techniques and analytical characterization is essential.

Three research articles focused on low and high energy electron beam. Depending on the kinetic energy of the electrons, an electron beam could be distinguished either as a high (HEEB; >300 keV) or a low energy electron beam (LEEB; ≤300 keV).

Hieke and Pillai and Bhatia and Pillai used HEEB with a 10 MeV, 15 kW eBeam linear accelerator. The first group of authors applied a lethal target dose of 7.0 kGy and investigated the associated sub-lethal injury with the potential VBNC states in *Escherichia coli*. After investigating the overall cellular functionality via analyzing bacteriophage infection, ATP level, metabolic activity, membrane integrity and DNA double-strand breaks; the authors concluded that the irradiated *E. coli* cells resembled viable, non-treated cells more closely than the thermal inactivated cells. After irradiation, the cells were still metabolically active up to 9 days.

Bhatia and Pillai investigated a similar research focus with inactivated pathogens *E. coli* 026:H11 and *Salmonella Typhimurium* after HEEB-inactivation and lethal doses of 2–3 kGy. The cells were noted as metabolically active even after the β-alanine, alanine, aspartate, and glutamate metabolic pathway analyses. Hence, they suggested the term “Metabolically Active yet Non-Culturable” for HEEB-inactivated bacterial cells.

Zhang et al. investigated the LEEB-based spore inactivation by using different energy levels of 80–200 keV and the energy inputs up to 9.8 kGy. LEEB, as an emerging non-thermal technology, can perform surface decontamination with less quality losses than the alternative thermal or chemical based treatments. The authors revealed that the appearance of LEEB-based inactivation efficiency is comparable to the other ionizing radiation techniques, like HEEB. However, the employed indicator for irradiation-based sterilization, *Bacillus pumilus* (DSM 492), was more sensitive than *Geobacillus stearothermophilus* (ATCC 7953) spores.

A technology with physical and chemical mode of action under investigation was cold atmospheric pressure plasma (CAPP). Waskow et al. applied a diffuse coplanar surface barrier discharge to inactivate the pathogens and fungal spores from the seeds. The seeds could germinate even after the CAPP treatment, upon which the seed surface was speculated to be sufficiently decontaminated. In terms of mechanisms, the authors could separate the effect of ultraviolet light from other plasma components and suggested physical damage to the cell envelope after advanced analysis of the treated *E. coli*.

Durek et al. focused on *Aspergillus niger* and *Penicillium verrucosum* (inoculated on barley) inactivation and the production of one of the most abundant food-contaminating mycotoxins, ochratoxin A (OTA). Under certain conditions, OTA levels increased even with reduced mold concentrations, possibly due to stress reactions that demonstrated the importance of mechanistic understandings and adapted process settings.

Besides, physical hurdles being the main focus of the Research Topic; innovative chemical hurdles such as natural antimicrobials, especially citral, carvacrol, (E)-2-hexenal, and thyme essential oils (EOs) were investigated by Braschi et al. The authors analyzed the morpho-physiological changes of *Listeria monocytogenes* Scott A and *E. coli* MG 1655 after different EO treatments by flow cytometry. They also developed a protocol to screen EO efficiency with different microorganisms.

Finally, biological hurdles were considered in the study of Li et al. who investigated the microbial community in primary dark tea during the pile-fermentation process.

Hence, to conclude the whole Research Topic, innovative MHT concepts are still not well-understood. So far, very limited combinations from a diverse hurdle portfolio have been applied. Mechanistic understanding of the novel MHT concepts is essential to leverage all the potential benefits and to produce higher quality, safe food products.

## Author Contributions

AM wrote the manuscript. All authors critically reviewed the manuscript for the intellectual content and approved the final version.

### Conflict of Interest Statement

The authors declare that the research was conducted in the absence of any commercial or financial relationships that could be construed as a potential conflict of interest.
